# Editorial: Responding to harm with compassion, accountability, and transparency

**DOI:** 10.3389/frhs.2026.1804296

**Published:** 2026-03-04

**Authors:** Evan M. Benjamin, Thomas H. Gallagher

**Affiliations:** 1 Department of Medicine, Harvard Medical School, Boston, MA, United States; 2 Health Policy and Management, Harvard TH Chan School of Public Health, Boston, MA, United States; 3 Core Faculty, Ariadne Labs, Harvard TH Chan School of Public Health and Brigham and Women’s Hospital, Boston, MA, United States; 4 Department of Medicine, Department of Bioethics and Humanities, University of Washington, Seattle, WA, United States; 5 Johns Hopkins Berman Institute of Bioethics, Johns Hopkins University, Baltimore, MD, United States

**Keywords:** communication, harm, resolution, risk, safety

## Introduction

In some respects, responding to patients who have been harmed by their care should be simple: Just tell them the truth about what happened. Or, put more bluntly, “When we mess up, we should fess up!” But over the last 25 years, it has become abundantly clear how difficult it is to turn this principle into highly reliable practice (1). Most harm is not due to the actions of an individual healthcare professional, but rather occurs when interprofessional healthcare teams encounter system failures. Furthermore, human nature strongly resists sharing information when something goes wrong. We minimize, rationalize, and hope if we keep our heads down, everything will blow over. Fear of litigation, reputational damage, and difficult encounters with upset patients and their families abound. The result? Patients and families often receive far less information and support following harm events than they would like, and organizational learning to prevent recurrences lags.

Yet despite these barriers, important progress has been made in programs for responding when patients are harmed by their healthcare, known generically as Communication and Resolution Programs (CRPs). The articles in this Special Research Topic both highlight challenges and point the way to future improvements.

Modern CRPs have their origins in efforts in the late 1990s to promote greater transparency in healthcare, both to improve patient safety but also in response to societal pressure for greater openness. Early “Disclosure and Apology” programs emphasized healthcare professionals and organizations making more proactive efforts to tell patients about harm events and apologize. Pioneering CRPs at the University of Michigan and others expanded this model to include making proactive offers of financial compensation when the harm was due to an error or system failure. Scholarly publications from these organizations and others that followed in their footsteps demonstrated that this approach could be implemented without a dramatic deterioration, and in some cases significant improvements, in liability experiences and costs (2–7). Expansion of this idea began to occur in other countries as well, such as the United Kingdom, Australia, and New Zealand (8).

Over time, the process for responding to harm in healthcare was refined to the point where CRPs are understood to include several key elements: (1) early harm event identification, (2) open, ongoing communication about what happened with the patient and family, (3) careful event analysis with a systems and quality improvement focus, (4) support for the involved patient, family, and healthcare professionals, and (5) proactive financial and non-financial resolution or reconciliation. In the US, the Centers for Medicare and Medicaid Services (CMS) as part of their Patient Safety Structural Measure (PSSM) recently began to require that all hospitals attest whether they have an evidence-based CRP, and whether they use standard metrics to track the progress of their CRP. Evidence about the effectiveness of CRPs, along with the challenge of inconsistent CRP implementation, continues to be published (9).

How can the field make progress towards a future where every patient harmed by their healthcare receives accountability, compassion, and transparency from the organization and professionals who provided this care? Four main themes emerge from the articles in this collection:

### Deepening patient and family involvement in the response to harm

The response to harm should be the most patient and family-centered element of healthcare, but far too often it is the least (10). The papers from O'Hara and from Ramsey describe the innovative Learn Together program in the United Kingdom for involving patients and families in incident investigations, as well as the challenges of achieving this goal O'Hara et al., Ramsey et al. Learn Together also highlights how the CRP process, despite good intentions, has the potential to inadvertently compound that harm patients and families are experiencing. Froding extends the notion of involving patients and families (as well as healthcare professionals) into harm investigations in the context of suicide, emphasizing how these perspectives are essential to process improvement and learning Froding et al. High-functioning CRPs actively seek out and learn from all who have been involved in harm events.

### Improving the CRP process will require more training, better language, and metrics

Commitment across healthcare to the general tenets of a CRP is high, but few organizations have developed highly reliably processes. To achieve reliability, we need CRP to be a systemic approach to safety improvement. Two papers from Grossniklaus emphasize the critical role of educating healthcare professionals, especially those in training, to participate effectively in the CRP process, and highlight how new technology can support this goal Grossniklaus, Addario et al., Grossniklaus, King et al. Benjamin reminds us that even some of the basic elements of CRPs, such as the language we use to describe these programs and how we interact with patients and families after harm, need to be much more patient-centered Benjamin et al. Shannon unpacks the complexity of what “apology” looks like in the CRP process and encourages us to think and train more broadly about this crucial CRP element Shannon et al. Metrics will be key to achieving the goal of highly reliable CRPs, as articulated by Sokol-Hessner et al. High-functioning CRPs apply principles of continuous improvement not only to the harm events they address, but also to the overall functioning of the program itself.

### Making good on the promise of CRPs as patient safety programs

CRPs are best seen as patient safety programs conducted in close collaboration with risk and claims management professionals, not efforts whose primary goal is reducing liability costs. Hickson demonstrates how the CRP process can shed light both on patient safety vulnerabilities as well as on lapses in professional conduct Hickson et al. Better understanding how CRPs can be more fully aligned with other institutional initiatives to analyze and respond to system problems, such as peer-review and patient complaints programs, will be important future directions for the field. The data that CRP programs collect can also shed light on patient safety challenges that may otherwise be hidden, such as Lodato's paper which uses CRP data to detect patient demographic factors associated with unexpected in-hospital patient deaths Lodato et al. High-functioning CRPs are proactive and lean into all of the information generated about harm and how it can be reduced.

### Increasing the involvement of legislators and regulators

Legislators, regulators, and national quality/safety organizations have taken multiple steps to encourage the development and use of CRPs, such as passing laws providing legal protections for information and apology shared with patients during the CRP process, formalizing state CRP/CANDOR programs and processes, and endorsing CRPs as a best practice. As above, in the United States the CMS PSSM and its requirement that hospitals attest whether they have a CRP represented a significant step forward by expressing the expectation of the largest US healthcare purchaser that reliable CRPs be used.

Yet for many stakeholders who are eager that CRPs be more widespread and effective, legal mandates and other enforceable national standards that require CRP implementation are appealing. Most countries have avoided creating legal mandates for CRPs. The UK and Australian represent notable exceptions, and both have enacted a statutory duty of CANDOR (SDC). Harrison reports on the benefits and challenges associated with implementing this legal requirement in Victoria, Australia Harrison et al. Their study noted the contribution that SDC made towards more highly reliable CRPs, but also significant challenges when trying to adapt rigid requirements to complex and variable CRP cases. Legal mandates also have the potential downside of leading to “check-the-box” CRP implementation, rather than the desired embrace of CRP as a core aspect of a healthcare organization's clinical mission. High-functioning CRPs will develop more quickly when legislators and regulators incentive the development of highly reliable CRPs and recognize those organizations with exemplary programs.

## Next steps

While the progress with CRPs is exciting, in many ways the field is only at the end of the beginning. Providing healthcare is a privilege granted by the public, one that brings with it the expectation that the profession will be self-regulating. To improve trust, CRPs need to be more patient-centered, have better training for health professionals, be implemented systematically and more consistently, use performance metrics, actually improve patient safety, and be properly regulated and encouraged. The articles in this Research Topic shed important light on the challenges and opportunities that lie ahead on the path to the development and implementation of highly reliable programs for responding to harm in healthcare with compassion, accountability, and transparency.
